# Associations between demographic, clinical, and socioeconomic factors and mental health in long COVID: A clinic-based cross-sectional study

**DOI:** 10.1371/journal.pone.0342516

**Published:** 2026-03-18

**Authors:** Anh N.Q. Pham, Julia Smith, Kaylee A. Byers, Kiffer G. Card

**Affiliations:** 1 Faculty of Health Sciences, Simon Fraser University, Burnaby, Canada; 2 College of Health Science Vin University, Hanoi, Vietnam; 3 School of Population and Public Health, University of British Columbia, British Columbia, Canada; University of Toronto, CANADA

## Abstract

**Background:**

Long COVID is associated with persistent symptoms, including the onset of new mental health challenges such as anxiety and depression, as well as the worsening of pre-existing conditions. While previous research has examined the impact of demographic factors, chronic conditions, and traumatic events on mental health, little is known about how these factors interact to shape mental health outcomes in individuals with Long COVID. This study investigates the relationship between selected demographics characteristics, social determinants of health – specifically living settings, place of living, employment status, and working hours – and chronic health conditions on mental health outcomes among individuals with Long COVID.

**Methods:**

This is a secondary analysis using previously collected survey data from 3,611 individuals, who were diagnosed, referred to and admitted at Post-COVID Recovery Clinics, British Columbia, Canada at the time of their admission. The dataset includes demographic variables (sex, age, living situation, employment status, occupation, ethnicity), history of chronic conditions, and mental health outcomes (anxiety and depression) of patients. Univariable and multivariable Generalized Linear Regression analyses were conducted to examine associations between SDoH and mental health outcomes, adjusting for potential confounders.

**Results:**

The cohort had a mean age of 50 years, and 62% of participants were female. Overall, 38% screened positive for anxiety, 35% for depression, and 26% for both conditions following COVID-19 infection. In multivariable analyses, younger age, cognitive issues, and activity limitations were significantly associated with symptoms of anxiety, while younger age, cognitive issues, and physical impairments were significantly associated with symptoms of depression. In the multivariable model, individuals with cognitive issues were more likely to report anxiety (RR = 1.45, 95% CI: 1.34–1.56) and depression (RR = 1.54, 95% CI: 1.42–1.68). Activity limitations were also associated with anxiety (RR = 1.12, 95% CI: 1.01–1.24), and physical impairments with depression (RR = 1.43, 95% CI: 1.24–1.65). Overall, 38% of patients reported symptoms of anxiety, 35% reported depression, and 26% experienced both following COVID-19 infection.

**Conclusion:**

These findings highlight important associations between mental health symptoms and clinical factors among individuals with Long COVID and underscore the need for further longitudinal research to clarify causal pathways and inform mental health support strategies, particularly among those with activity limitations, cognitive and physical impairments.

**Patient or public contribution:**

The data collection and questionnaire were designed and conducted by the Post-COVID Integrated Clinic Network in British Columbia, Canada.

## Introduction

Long COVID, also known as post-acute sequelae of SARS-CoV-2 infection, is characterized by the persistence of symptoms and health impairments well beyond the acute phase of COVID-19. Among its wide-ranging effects, the condition is frequently associated with substantial mental health challenges, including anxiety and depression [1]. These challenges are often exacerbated by the loss of autonomy, the need for caregiving support, reduced capacity to perform daily tasks, and profound changes to life roles and personal identity. These mental health outcomes not only diminish quality of life but also impose significant burdens on healthcare systems and affected individuals [2]. Systematic reviews and meta-analyses have reported substantial prevalence of anxiety and depressive symptoms among individuals with Long COVID/post-COVID conditions, although estimates vary by population, measurement tools, and timing since infection. For example, recent evidence syntheses have consistently identified depression and anxiety among the more frequently reported longer-term sequelae, often alongside fatigue, sleep disturbance, and cognitive complaints [1].

Research highlights that demographic factors such as age, sex, and socioeconomic status significantly influence mental health outcomes [3,4]. Growing evidence also points to the role of social determinants of health (SDoH) – including housing stability, employment conditions, social support, and neighborhood environment – in relation to these outcomes among individuals with Long COVID. Socioeconomic disadvantage, job insecurity, and social isolation have been linked to heightened psychological distress and delayed recovery [5,6], with financial strain and unstable housing contributing to greater anxiety and depression through cumulative stress and limited access to resources [7]. These inequities in living and working conditions may further compound existing health disparities, amplifying the mental health burden in marginalized populations [8]. In parallel, chronic conditions, including preexisting psychiatric or neurological disorders, have been identified as predictors of vulnerability [9,10]. However, while prior work has described mental health burden in Long COVID, fewer studies have simultaneously examined how socioeconomic and living/working circumstances co-occur with clinical limitation profiles (e.g., cognitive, physical, and activity limitations) associate with anxiety and depression among patients presenting for care—particularly in Canadian clinic-based cohorts [11,12]. Understanding these interactions is essential for tailoring interventions and support strategies to address the unique challenges faced by this population.

This study, informed by a social determinants of health framework [13] and an intersectional perspective [14], seeks to address this gap by examining the interplay between demographic factors, and chronic conditions in relation to mental health outcomes among individuals with Long COVID. In the context of Long COVID, cognitive and physical impairments may intersect with socioeconomic factors such as employment and living arrangements to influence anxiety and depression. These frameworks guided variable selection and interpretation of findings in this clinic-based analysis.

The objectives of this study was to examine the prevalence of anxiety and depressive symptoms among individuals with Long COVID referred to Post-COVID Recovery Clinics in British Columbia, and to assess the associations between demographic characteristics, social determinants of health, and clinical limitation profiles and the presence of anxiety and depressive symptoms. We hypothesized that [1] symptoms of anxiety and depression would be highly prevalent in this clinic-based Long COVID population, and [2] cognitive, physical, and activity-related limitations, as well as selected socioeconomic factors, would be associated with a higher likelihood of anxiety and depressive symptoms.

## Methods

### Study design and setting

This study was conducted within the Post-COVID-19 Interdisciplinary Clinical Care Network (PC-ICCN) in British Columbia, Canada. The PC-ICCN is a provincially coordinated initiative that aims to support optimal recovery among individuals experiencing persistent symptoms following SARS-CoV-2 infection through integrated clinical care, education, and research. The Network is a partnership involving the provincial Ministry of Health, the Provincial Health Services Authority, Providence Health Care (which manages the provincial virtual clinic), regional health authorities, patients, and research organizations across the province [15].

Clinical care is delivered through a province-wide virtual Post-COVID Recovery Clinic serving individuals throughout British Columbia. The clinic operates as an interdisciplinary, rehabilitation-focused service staffed by allied health professionals, nurses, physicians, and other team members, with an emphasis on education and self-management strategies for people living with Long COVID. Patients are referred by primary care providers and complete a standardized intake questionnaire at the time of clinic admission; admission to the clinic therefore served as a pragmatic proxy for Long COVID status. Survey responses were documented in the Patient Records Outcomes and Information System (PROIS), a province-wide data platform managed by the Provincial Health Services Authority (PHSA) [16]. The present analysis included all patients who completed the questionnaire at their initial admission between July 2020 and November 2023.

The complete questionnaire is provided in Supplementary 1, with this study focusing on survey sections related to social demographics, medical history and comorbidities, and results from anxiety and depression screening. The questionnaire did not include standardized measures of symptom severity; however, it captured the presence of cognitive, physical, sensory, and activity-related limitations, as well as the timing of initial COVID-19 infection.

### Data access

The dataset was accessed in February 2025. The research team did not have access to identifiable information at any time point during or after the data collection.

### Variables of Interest

The primary dependent variable in this study was mental health outcomes, assessed through measures of anxiety and depression symptomology using validated scales:

Symptoms of anxiety was evaluated using the Generalized Anxiety Disorder-2 (GAD-2) scale, a validated screening instrument with demonstrated good sensitivity (76–86%) and specificity (81–83%) for generalized anxiety disorder with a cut-off score of 3. Participants responded to two questions: *“How often have you been feeling nervous, anxious, or on edge?”* and *“How often have you not been able to stop or control your worrying?”* [17].

Symptoms of depression was assessed using the Patient Health Questionnaire-2 (PHQ-2), a widely used and validated screening tool with sensitivity ranging from 79–87% and specificity from 77–92% for major depressive disorder also with a cut-off score of 3. This measure included the questions: *“How often have you experienced little interest or pleasure in doing things?”* and *“How often have you been feeling down, depressed, or hopeless?”* [18].

The analysis includes a range of variables categorized into demographic factors and existing chronic conditions. Demographic characteristics encompass sex, age, while social determinants of health include ethnicity, living settings, place of living, employment status, and working hours. Pre-existing conditions are divided into several subcategories: cognitive and neurological issues (dementia, cognitive problems, stroke effects, epilepsy); sensory impairments (cataracts, glaucoma, vision problems, and hearing issues); physical functioning issues (difficulties with walking, dexterity, and speech, chronic pain that restricts activities); activity limitations and assistance needs (reduced activity levels at home, work, or school and the need for help with daily tasks); and chronic conditions and comorbidities (asthma, arthritis or rheumatism, back problems (excluding arthritis), high blood pressure, migraine headaches, chronic bronchitis or emphysema, diabetes, heart disease, cancer, stomach or intestinal ulcers, and urinary incontinence). These variables capture the complexity of health challenges faced by individuals and their cumulative impact on well-being.

### Data analysis

As this study used all available eligible participants from an existing clinical dataset, no formal a priori sample size calculation was performed. Statistical methods, including descriptive and inferential analyses, were used to examine relationships between variables. A purposeful variable selection approach was used for multivariable modeling. Candidate variables were identified a priori based on clinical relevance and prior literature, and univariable analyses were used to assess initial associations with anxiety and depression outcomes. Variables demonstrating meaningful associations were considered for inclusion in multivariable models. Multicollinearity among independent variables was assessed prior to final model specification, and highly correlated variables were excluded to improve model stability and interpretability. Since the outcomes (anxiety and depression) are binary with high prevalence, a generalized linear model (GLM) with a Poisson family was used to estimate relative risks (RRs) with 95% confidence interval (CI). It provides a more accurate and interpretable estimate of relative risks for binary outcomes with high prevalence, while reducing the bias associated with other regression in this context [19]. The inferential approach enabled us to capture the combined effects of multiple social determinants on mental health outcomes through stratified analyses or interaction terms [14]. Missing data were handled by exclusion.

We also conducted an analysis of the correlations and associations among the independent variables to identify potential multicollinearity issues [20]. Variables that exhibited high collinearity—meaning their inclusion could distort the results or inflate the variance of the regression estimates—were excluded from the final model. This step was crucial to ensure the robustness and interpretability of the model’s results by minimizing redundancy and improving the precision of the remaining variables.

All analyses were conducted using Python 3.11.8 [21]

### Ethics declaration

This study has received approval from the Research Ethics Board at the Simon Fraser University (#30001542) and adheres to all relevant guidelines and regulations for research involving de-identified health data (e.g., CHREB, Tri-Council Policy Statement on the Ethical Conduct for Research Involving Humans [TCPS2]).

### Participant consent

According to the Freedom of Information and Protection of Privacy Act—British Columbia Law (RSBC 1996, c 165, “https://canlii.ca/t/566l3” retrieved on 2024-07-22), aggregated data from medical records can be used for research purposes at health organizations; therefore, informed consent is not required.

## Findings

### Sociodemographic and clinical characteristics of participants

The analysis includes 3,613 patients with Long COVID who completed the questionnaire at admission. After excluding those with missing data on age, sex, and EQ-VAS, 3,611 patients were included. The mean age was 50 years (SD: 15), with 48% aged 40–59. Among the respondents, 62% were female, 59% were Caucasian, 28% were Asian, and smaller percentages identified as Indigenous (3%), Latin American (2%), and Black (1%). Further details on the cohort characteristics are available elsewhere [22].

Applying a cut-off of three points for positive diagnosis of anxiety and depression [23,24], 38% of our cohort experienced symptoms of anxiety and 35% experienced symptoms of depression. We present prevalence of anxiety and depression across groups of demographic and socioeconomic characteristics (Table 1), as well as pre-existing clinical conditions (Table 2).

**Table 1:** Prevalence of anxiety and depression (and both) among people with Long COVID by demographic characteristics.

Characteristic	Group	Total	Anxiety	Depression
		3611 (100%)	1382 (38%)	1249 (35%)
**Sex**	Female	2233 (62%)	935 (42%)	800 (36%)
	Male	1378 (38%)	447 (32%)	449 (33%)
**Living settings**	Live alone	655 (18%)	296 (45%)	271 (41%)
	Live with other(s)	2781 (77%)	1060 (38%)	956 (34%)
**Place of living**	A condo or apartment	1007 (28%)	428 (43%)	394 (39%)
	A house or townhouse or mobile home	2356 (65%)	906 (38%)	809 (34%)
	An assisted-living facility	18 (0%)	5 (28%)	7 (39%)
**Employment status**	Unemployed	1046 (29%)	392 (37%)	370 (35%)
	Employed	2371 (66%)	952 (40%)	848 (36%)
**Working hours**	Work full time (regular hours)	1235 (34%)	438 (35%)	415 (34%)
	Work part time (greater than 50% of regular hours)	267 (7%)	108 (40%)	87 (43%)
	Work part time (less than 50% of regular hours)	260 (7%)	107 (41%)	94 (36%)
	Unable to work following COVID-19 illness	575 (16%)	288 (50%)	247 (33%)
**Ethnicity**	Asian	797 (22%)	237 (30%)	226 (28%)
	Black (e.g., African, Somali)	25 (1%)	11 (44%)	10 (40%)
	Indigenous (formerly Aboriginal)	118 (3%)	53 (45%)	55 (47%)
	Latin American	86 (2%)	29 (34%)	24 (28%)
	White (Caucasian)	201 (6%)	89 (44%)	86 (43%)
	Others	2082 (58%)	897 (43%)	784 (38%)

**Table 2:** Prevalence of anxiety and depression (and both) among people with Long COVID by pre-existing condition.

Condition(s)	Group	Total	Anxiety	Depression
**Cognitive issue**	No issue	1571 (43%)	261 (68%)	217 (66%)
	One issue	1105 (31%)	482 (44%)	417 (38%)
	Two or more issues	937 (26%)	639 (17%)	616 (14%)
**Sensory impairment**	No issue	2068 (57%)	692 (33%)	613 (30%)
	One issue	1035 (29%)	447 (43%)	416 (40%)
	Two or more issues	510 (14%)	243 (48%)	221 (43%)
**Physical issues**	No issue	551 (15%)	39 (48%)	32 (44%)
	One issue	452 (13%)	100 (22%)	80 (18%)
	Two or more issues	2610 (72%)	1243 (7%)	1138 (6%)
**Activity limitations**	No issue	777 (22%)	96 (12%)	85 (11%)
	One issue	804 (22%)	316 (39%)	298 (37%)
	Two or more issues	2032 (56%)	970 (48%)	867 (43%)
**Chronic conditions**	No issue	664 (18%)	158 (24%)	130 (20%)
	One issue	744 (21%)	274 (37%)	233 (31%)
	Two or more issues	2205 (61%)	950 (43%)	887 (40%)

### Prevalence of outcomes

**Fig 1:**
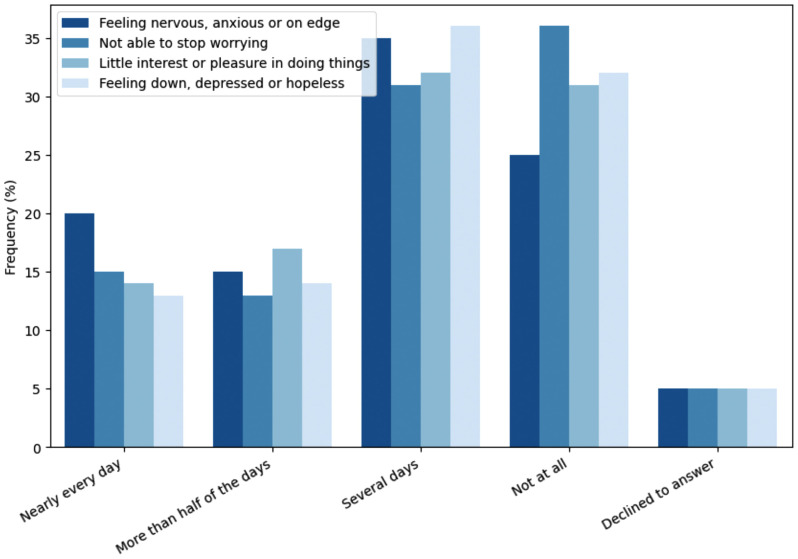
Frequency of anxiety and depression in patients with Long-COVID-19.

Fig 1 illustrates responses to four anxiety-related questions, categorized by frequency of the condition, across several groups. The general trend across the four questions shows that about half of individuals (n = 3,613) report experiencing these feelings of anxiety, depression, and worry less frequently (i.e., “Not at all” or “Several days”), while the other half of respondents show significant distress, as indicated by higher counts in categories like “More than half of the days” or “Several days”.

### Factors associated with anxiety and depressive symptoms

In the univariate analysis, age, sex, work status (full-time vs. part-time), cognitive issues, sensory issues, physical issues, activity limitations, and chronic conditions were significantly associated with anxiety (p ≤ 0.001). The significant associations suggest that these demographic and work-related factors are important in understanding anxiety risk in the unadjusted analysis.

**Fig 2:**
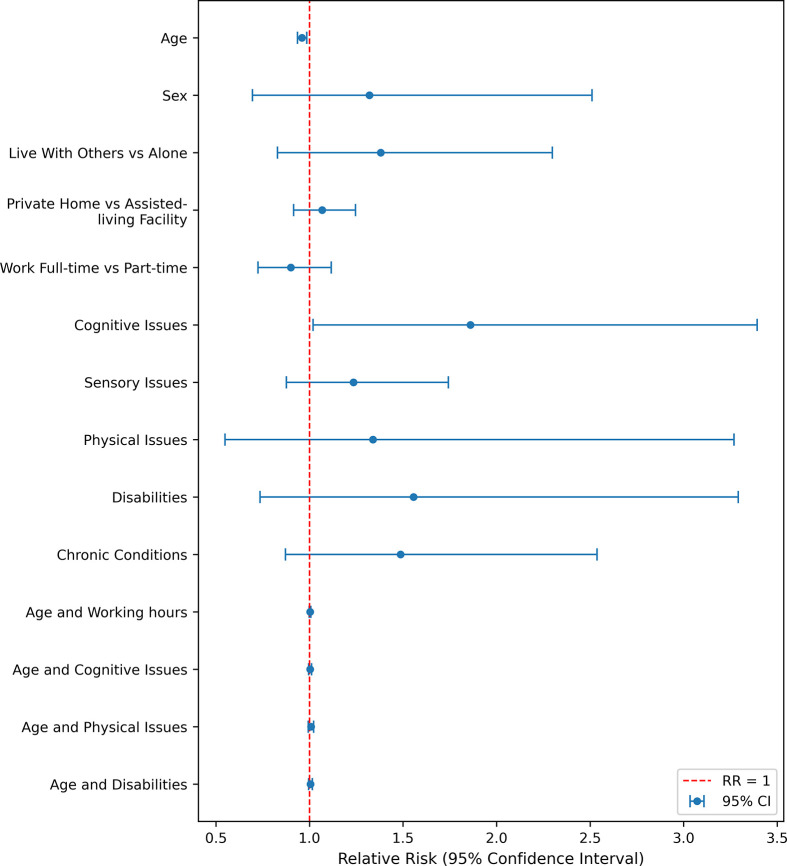
Forest Plot of Relative Risks for Anxiety among people with Long COVID, BC-ICCN 2020–2023.

However, when adjusting for other factors in the final model, only age (RR = 0.902, 95% CI: 0.840–0.969, p = 0.005), cognitive issues (RR = 1.447, 95% CI: 1.339–1.562, p = 0.000), and activity limitations (RR = 1.118, 95% CI: 1.012–1.235, p = 0.029) remained significant (Fig 2). This suggests that older individuals were slightly less likely to experience symptoms of anxiety. While individuals with activity limitations and cognitive issues are more likely to experience anxiety compared to the those without.

**Fig 3:**
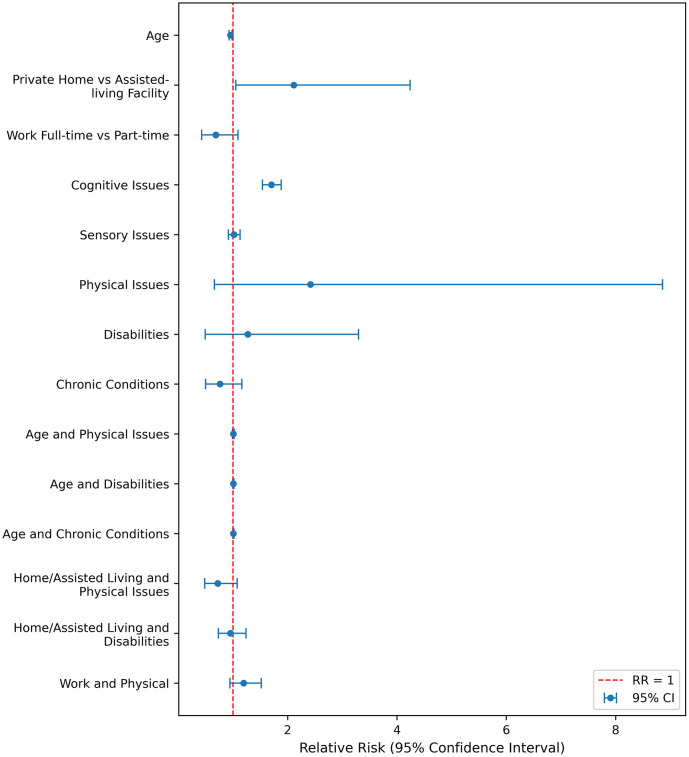
Forest Plot of Relative Risks for Depression among people with Long COVID, BC-ICCN 2020–2023.

Univariate analysis exploring depression showed significant associations for age, work status, cognitive issues, sensory issues, physical issues, activity limitations, and chronic conditions (p ≤ 0.005). However, in the multivariable analysis, only age (RR = 0.915, 95% CI: 0.849–0.987, p = 0.021), cognitive issues (RR = 1.543, 95% CI: 1.421–1.675, p = 0.000), and physical issues (RR = 1.427, 95% CI: 1.236–1.647, p = 0.000) remained significantly associated with depression. Other variables did not retain significance after adjustment (Fig 3). This suggests that older age people are also less likely to experience symptoms of depression while pre-existing cognitive and physical issues link to an increase in the likelihood of experiencing depression. Other variables, including sensory issues, physical issues, and chronic conditions, while significant in the univariable analysis, were not significant in the multivariable model. The insignificance in the multivariable analysis indicates that their association with depression might be more complex or conditional.

Complete tables reporting p-values and RR from univariable and multivariable logistic regression are included in Supplementary 2.

## Discussion

This study provides insights into the social determinants of health and their relationship with mental health outcomes—specifically anxiety and depression—among individuals with Long COVID. Our cohort, consisting of 3,611 patients with diverse demographic backgrounds, underscores the significant mental health burden associated with Long COVID. Specifically, 38% of participants experience anxiety symptoms, 35% report depression symptoms, and 26% experience both. These figures are five to seven times higher than the prevalence rates reported by Statistics Canada for the general population, which are 5.2% for anxiety and 7.6% for depression [25]. However, these findings align with existing literature, underscoring the psychological toll of prolonged illness and the intersectional influence of social determinants [1]. While it is important to note that anxiety and depressive symptoms in this study were identified using brief screening instruments rather than diagnostic interviews, and therefore represent probable symptoms rather than confirmed clinical diagnoses, these findings warrant closer examination of the underlying social and clinical factors, as well as their consistency with prior research on COVID-19 and mental health..

### Social economic factors and mental health

Although the associations between living settings, work arrangements, and multigenerational living were not statistically significant in our analysis, the trends observed in mental health symptoms merit attention. For instance, individuals who live alone exhibited a higher prevalence of anxiety and depression compared to those living with others, which suggests that social isolation could be a significant risk factor. Although no studies have specifically investigated the relationship between living in an assisted-living facility or long-term care and mental health outcomes in individuals with Long COVID, similar trends have been observed in those with acute COVID-19. In these cases, residing in less isolated settings was found to be beneficial for mental health, suggesting that the environment plays a crucial role in managing depressive symptoms during recovery from COVID-19 [26]. Additionally, while the intersection of lower mental health prevalence among Asian and Latin American groups was not statistically significant, it is worth considering how living in multigenerational households might act as a protective factor. For instance, one-third of Latin Americans in the U.S. live in multigenerational homes, which may contribute to lower rates of mental health symptoms due to access to supports in the home [27]. This finding could support the potential benefit of community-based interventions designed to connect people, particularly those who live alone, with social and mental health resources.

The trend of more popular mental health symptoms among those unable to work after COVID-19 illness is also noteworthy. The added anxiety stemming from the inability to work and limited financial, or support resources could explain this trend. Though not significant in this analysis, this warrants further investigation into the potential benefits of supportive and adapting work arrangements for mental well-being.

### Clinical history and mental health

The combined variable for activity limitations showed a significant association with anxiety (RR = 1.1), aligning with previous research indicating that activity limitations and assistance needs—such as reduced activity levels at home, work, or school and the need for help with daily tasks—are linked to increased anxiety and depression in the general population [28]. As expected, cognitive issues demonstrated a strong positive association with both anxiety (RR = 1.4) and depression (RR = 1.5), while physical issues were significantly associated with depression (RR = 1.4). These findings are consistent with longstanding evidence that individuals with cognitive impairments, such as dementia, face a heightened risk of depression [29]. Managing multiple health conditions simultaneously may exacerbate mental health challenges, and the emotional strain of coping with chronic illness—coupled with the psychological burden of uncertainty—can further contribute to depression [30]. These results highlight the critical need to address mental health concerns early in patients with Long COVID, particularly those with cognitive and physical impairments. Targeted interventions to support mental well-being in these populations could play a key role in preventing the onset or worsening of depression.

### Anxiety and depression

Anxiety and depression are well-known to frequently co-occur [31]. One of the most significant findings in this study is the strong bidirectional relationship between anxiety and depression. Depression emerged as a significant associated factors of anxiety, with an RR of 4.4, even after adjusting for other variables. Similarly, anxiety significantly associated with depression, with an RR of 8.4, indicating a robust link between these two mental health conditions. This bidirectional relationship suggests that interventions targeting one condition may benefit the other, emphasizing the need for integrated mental health care in Long COVID clinics.

### Limitations of the study

This study has several limitations that should be acknowledged. First, the data were collected at the time of admission to Long COVID clinics, which represents a single time point and may not capture changes in mental health outcomes over time. Longitudinal data would provide a more comprehensive understanding of the trajectory of anxiety and depression in this population. Second, mental health outcomes were assessed using brief, validated screening instruments (GAD-2 and PHQ-2) rather than structured diagnostic interviews. While these tools are widely used and demonstrate good psychometric performance, they identify probable anxiety and depressive symptoms with possibility of recall bias and social desirability bias and should not be interpreted as clinical diagnoses. Consequently, prevalence estimates may differ from those based on diagnostic assessments. Third, as our cohort was drawn from patients actively seeking care at Long COVID clinics, potentially excluding individuals with less severe symptoms or those facing barriers to healthcare access. As a result, the findings may not fully represent the broader Long COVID population, particularly those not engaging with formal healthcare services. Forth, the regression models included a selected set of demographics, socioeconomic, and clinical variables and are not exhaustive. Important factors such as social support, access to mental health services, prior mental health history, symptom severity, and longitudinal changes were not available in the dataset and therefore could not be included. As a result, residual confounding is possible. Lastly, given the cross-sectional nature of this study, the observed relationships should be interpreted as associations rather than causal effects, and bidirectional relationships between mental health symptoms and clinical or social factors are likely.

### Implications for policy and practice

Understanding how these factors interact provides a foundation for developing targeted interventions and informing policies that address the mental health burden of Long COVID. Given the high prevalence of anxiety and depression among individuals with Long COVID, integrating mental health screening into routine care is essential. Expanding access to mental health services, particularly for underserved communities, is critical to addressing these needs. Current funding for mental health support must be reassessed, as the cost of care often exceeds affordability for individuals without health insurance—especially those unable to work due to Long COVID. Moreover, the strong interconnection between anxiety and depression underscores the importance of early intervention strategies to prevent the compounding effects of co-occurring conditions. Policymakers should prioritize funding for interdisciplinary clinics and community-based initiatives that provide equitable, comprehensive care for Long COVID patients.

The significant associations between mental health outcomes and social determinants, such as living arrangements, emphasize the need for a holistic approach that addresses both medical and social factors. Programs offering support for employment and housing stability can help alleviate the mental health burden among those facing socioeconomic challenges. Importantly, these actions don’t just support Long COVID patients, but all of society and take a muli-solving approach to community wellbeing. This discussion also highlights the importance of reimagining urban planning to foster supportive communities. For instance, designing neighborhoods with shared resources and social structures – similar to “retirement community” models – could benefit individuals living alone by providing built-in support systems.

### Future research directions

Future longitudinal studies are needed to track changes in mental health outcomes over time and to explore causal relationships between SDoH and mental health conditions. Additionally, research should focus on identifying effective interventions tailored to the unique needs of individuals with Long COVID, particularly those from vulnerable populations. More comprehensive data collection, including objective measures of mental health and social determinants, could enhance the validity of findings. Further investigation into the mechanisms underlying the observed associations, such as the interplay between mental health and physical comorbidities, would provide valuable insights. Future studies should also explore the role of access to healthcare services, including mental health care, and examine disparities in outcomes based on race, ethnicity, and socioeconomic status. These efforts could inform targeted policies and interventions to improve the well-being of Long COVID patients.

## Conclusion

This study highlights a substantial burden of self-reported anxiety and depressive symptoms among individuals with Long COVID, as identified through validated screening tools. Anxiety and depression were highly prevalent and strongly interrelated, underscoring the need for integrated mental health care within Long COVID services. Significant associations were observed between mental health symptoms, SDoH and clinical history – particularly living arrangements and physical and cognitive impairments – which reflect the broader social and structural contexts shaping recovery. Although some associated factors, such as employment and ethnicity, were not statistically significant, their consideration remains important for understanding patterns of vulnerability and resilience within diverse populations. Overall, these findings align with the study’s objective to examine how demographic, social, and clinical factors interact with mental health in Long COVID, emphasizing the need for coordinated clinical and policy responses that address both individual and social determinants of well-being.

### Key messages

- High Prevalence of Mental Health Challenges – Anxiety (38%) and depression (35%) are common among individuals with Long COVID, with 26% experiencing both conditions.
- Older age is associated with a lower risk of both anxiety (RR = 0.902) and depression (RR = 0.915) in Long COVID patients.
- Cognitive issues significantly linked to higher risk of both anxiety (RR = 1.447) and depression (RR = 1.543), while physical issues are strongly linked to depression (RR = 1.427).
- Findings underscore the importance of addressing cognitive and physical impairments when designing mental health interventions for Long COVID patients.

## Supporting information

S1 File Survey questionnaire. (PDF)

S2 File Findings. (DOCX)
