# Supplementary material for: Associations between demographic, clinical, and socioeconomic factors and mental health in long COVID: A clinic-based cross-sectional study
Source: PLoS One. 2026 Mar 18;21(3):e0342516. doi: 10.1371/journal.pone.0342516 (PMC12998830; doi:10.1371/journal.pone.0342516)
Supplement: S1 File — (PDF) [file pone.0342516.s001.pdf]

Print | Clear Form

POST COVID-19 RECOVERY CLINIC
BASELINE QUESTIONNAIRE

Questionnaire completed on: (dd/mmm/yyyy) ____________________

Clinic visit date: (dd/mmm/yyyy) __________________________ *(if different from completion date)*

1. PATIENT DEMOGRAPHICS

Name: ________________________________ ________________________ ______________
Last *First* *Middle*

Date of Birth: (dd/mmm/yyyy) ________/________/__________

What is your living situation? Please check ONE answer for each question.

I live:
- ☐ Alone
- ☐ With other(s)

My primary residence (home) is:
- ☐ A house or townhouse or mobile home
- ☐ A condo or apartment
- ☐ An assisted-living facility
- ☐ I do not have a home

Are you employed? ☐ Yes ☐ No

If Yes, what is your occupation: ____

Industry of employment: ____

Do you work:
- ☐ Full time (regular hours)
- ☐ Part time (greater than 50% of regular hours)
- ☐ Part time (less than 50% of regular hours)
- ☐ Unable to work following COVID-19 illness

What is your ethnicity?
- ☐ White (Caucasian)
- ☐ Indigenous
- ☐ Black (e.g. African, Somali)
- ☐ Latin American
- ☐ Asian: (specify) ______________________________
- ☐ Other: (specify) ______________________________

2. SMOKING HISTORY

Have you ever smoked cigarettes? ☐ Yes ☐ No

If Yes: Do you smoke cigarettes now (at least 1 per day for the past year)? ☐ Yes ☐ No

What year did you start smoking? ___________________

What year did you stop smoking? _________ (if you are still smoking mark N/A) ☐ N/A

On average, how any cigarettes do/did you smoke per day? _____________________

Did you use vaping products in the 3 months before you had COVID-19? ☐ Yes ☐ No

Did you smoke marijuana in the 3 months before you had COVID-19? ☐ Yes ☐ No

POST-COVID-19 RECOVERY CLINIC
BASELINE QUESTIONNAIRE

2. COVID-19 HISTORY

**Which of the following symptoms were new or worse than usual when you had COVID-19?**	New or worse symptoms just before or during admission to hospital	Symptoms still present
Shortness of breath	☐	☐
Cough	☐	☐
Sore throat	☐	☐
Chest congestion (phlegm production)	☐	☐
Chest pain	☐	☐
Palpitations	☐	☐
Headache	☐	☐
Fever	☐	☐
Fatigue	☐	☐
Weakness	☐	☐
Loss of taste and smell	☐	☐
Hoarse voice/change in voice	☐	☐
Nausea or vomiting	☐	☐
Diarrhea	☐	☐
Muscle or joint aches	☐	☐
Rashes	☐	☐
Discolouration of fingers or toes	☐	☐
Other (specify):	☐	☐

When did your first symptom(s) start? (dd/mmm/yyyy) ________/________/__________

Do your symptoms fluctuate in severity? ☐ Yes ☐ No

Are your symptoms brought on by: ☐ Physical exertion ☐ Cognitive tasks ☐ Emotional events

Do your symptoms improve with rest? ☐ Yes ☐ No

3. MEDICAL STATUS

Please answer "Yes" or "No" for all the items below as they apply to you now.
If you are unsure, please select the option that feels most appropriate to you.

Long term disabilities or handicaps	☐ Yes	☐ No
Restriction of activity due to poor health	☐ Yes	☐ No
Help for preparing meals	☐ Yes	☐ No
Help for shopping for necessities	☐ Yes	☐ No
Help for housework	☐ Yes	☐ No
Help for heavy household chores	☐ Yes	☐ No
Help for personal care	☐ Yes	☐ No
Help for moving inside house	☐ Yes	☐ No
Food allergies	☐ Yes	☐ No
Asthma	☐ Yes	☐ No

POST COVID-19 RECOVERY CLINIC
BASELINE QUESTIONNAIRE

3. MEDICAL STATUS (continued)

Arthritis or rheumatism	☐ Yes	☐ No
Back problems other than arthritis	☐ Yes	☐ No
High blood pressure	☐ Yes	☐ No
Migraine headaches	☐ Yes	☐ No
Chronic bronchitis or emphysema	☐ Yes	☐ No
Diabetes	☐ Yes	☐ No
Epilepsy	☐ Yes	☐ No
Heart disease	☐ Yes	☐ No
Cancer	☐ Yes	☐ No
Stomach or intestinal ulcers	☐ Yes	☐ No
Effects of a stroke	☐ Yes	☐ No
Problems with urinary incontinence	☐ Yes	☐ No
Dementia	☐ Yes	☐ No
Cataracts	☐ Yes	☐ No
Glaucoma	☐ Yes	☐ No
Other chronic conditions	☐ Yes	☐ No
Vision problem	☐ Yes	☐ No
Hearing problem	☐ Yes	☐ No
Walking difficulty	☐ Yes	☐ No
Dexterity problem	☐ Yes	☐ No
Cognitive problem	☐ Yes	☐ No
Body pain that prevents activities	☐ Yes	☐ No
Speech problem	☐ Yes	☐ No
Depression	☐ Yes	☐ No
Difficulty carrying light weight	☐ Yes	☐ No
Unhappy	☐ Yes	☐ No
Less activity at home, work, or school	☐ Yes	☐ No
Feeling hopeless	☐ Yes	☐ No
Lost weight	☐ Yes	☐ No
Self-reported poor health	☐ Yes	☐ No
Difficulty with problem solving	☐ Yes	☐ No
Always feeling tired	☐ Yes	☐ No

POST-COVID-19 RECOVERY CLINIC
BASELINE QUESTIONNAIRE

4. COUGH

Do you have a cough?

This includes any chronic cough, even if there is no phlegm or mucus. Do not include clearing your throat.

☐ No ☐ Yes – If Yes, what date did the cough start? (*dd/mmm/yyyy*) ______/______/__________

Place a vertical mark across the line below to indicate the severity of your cough:

No cough |_______________|______________________________| **Worst cough imaginable**

Do you cough up phlegm or mucus? ☐ Yes ☐ No

5. SHORTNESS OF BREATH

For each activity listed below, please rate your breathlessness on a scale of 0 to 5, where 0 is not at all breathless and 5 is maximally breathless or too breathless to do the activity. If the activity is one which you do not perform, please give your best estimate of breathlessness. Your responses should be for an average day during the past week.

PLEASE RESPOND TO ALL ITEMS.

How short of breath do you get while:	Not at all breathless 0	1	2	3	4	Maximally breathless or too breathless to do the activity 5
At rest	☐	☐	☐	☐	☐	☐
Walking on level surface at your own pace	☐	☐	☐	☐	☐	☐
Walking on level surface with others your age	☐	☐	☐	☐	☐	☐
Walking up a hill	☐	☐	☐	☐	☐	☐
Walking up stairs	☐	☐	☐	☐	☐	☐
While eating	☐	☐	☐	☐	☐	☐
Standing up from a chair	☐	☐	☐	☐	☐	☐
Brushing your teeth	☐	☐	☐	☐	☐	☐
Shaving and/or brushing hair	☐	☐	☐	☐	☐	☐
Showering/bathing	☐	☐	☐	☐	☐	☐
Dressing	☐	☐	☐	☐	☐	☐
Picking up and straightening	☐	☐	☐	☐	☐	☐
Doing dishes	☐	☐	☐	☐	☐	☐
Sweeping/vacuuming	☐	☐	☐	☐	☐	☐
Making the bed	☐	☐	☐	☐	☐	☐
Shopping	☐	☐	☐	☐	☐	☐
Doing laundry	☐	☐	☐	☐	☐	☐
Washing the car	☐	☐	☐	☐	☐	☐
Mowing the lawn	☐	☐	☐	☐	☐	☐
Watering the lawn	☐	☐	☐	☐	☐	☐
Sexual activities	☐	☐	☐	☐	☐	☐

How much do the things below limit you in your daily life?	Does not limit me					Completely limits me
Shortness of breath	☐	☐	☐	☐	☐	☐
Fear of hurting myself by overexerting	☐	☐	☐	☐	☐	☐
Fear of shortness of breath	☐	☐	☐	☐	☐	☐

POST COVID-19 RECOVERY CLINIC
BASELINE QUESTIONNAIRE

6. QUALITY OF LIFE

Under each heading, tick ONE box that best describes your health TODAY.

MOBILITY
- ☐ I have no problems walking about
- ☐ I have slight problems walking about
- ☐ I have moderate problems walking about
- ☐ I have severe problems walking about
- ☐ I am unable to walk about

SELF-CARE
- ☐ I have no problems washing or dressing myself
- ☐ I have slight problems washing or dressing myself
- ☐ I have moderate problems washing or dressing myself
- ☐ I have severe problems washing or dressing myself
- ☐ I am unable to wash or dress myself

USUAL ACTIVITIES *(e.g. work, study, housework, family or leisure activities)*
- ☐ I have no problems doing my usual activities
- ☐ I have slight problems doing my usual activities
- ☐ I have moderate problems doing my usual activities
- ☐ I have severe problems doing my usual activities
- ☐ I am unable to do my usual activities

PAIN / DISCOMFORT
- ☐ I have no pain or discomfort
- ☐ I have slight pain or discomfort
- ☐ I have moderate pain or discomfort
- ☐ I have severe pain or discomfort
- ☐ I have extreme pain or discomfort

ANXIETY / DEPRESSION
- ☐ I am not anxious or depressed
- ☐ I am slightly anxious or depressed
- ☐ I am moderately anxious or depressed
- ☐ I am severely anxious or depressed
- ☐ I am extremely anxious or depressed

➔ **To move the X on this scale:** Place your cursor in front of the "X" and click return on your keyboard until the X is in position next to the number you want to record.

The best health you can imagine

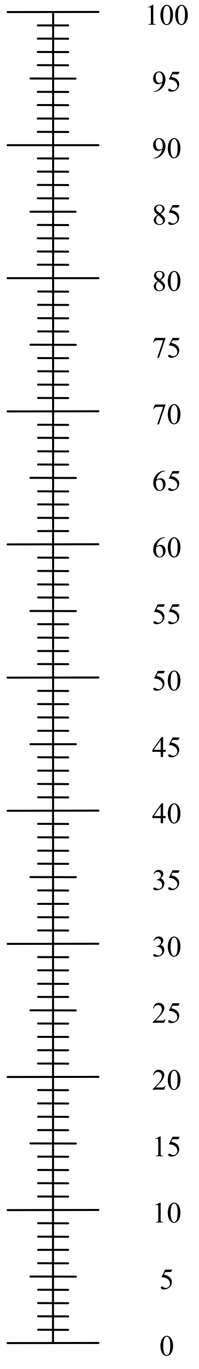

The worst health you can imagine

We would like to know how good or bad you feel your health is TODAY.

The scale to the right, is numbered from 0 to 100.

100 means the <u>BEST</u> health you can imagine.

0 means the <u>WORST</u> health you can imagine.

Mark an X on the scale to indicate how you feel your health is TODAY.

Now, please write the number you marked on the scale in the box below.

YOUR HEALTH TODAY = ☐

7. NEUROLOGY SCREEN

**Have you experienced any of the following symptoms?**	**Symptom present before COVID illness**	**Symptom new or much worse during acute COVID illness**	**Symptom experienced at present**
Weakness in face, arms, or legs (or all)	☐	☐	☐
Numbness/loss of feeling in face, arms, or legs (or all)	☐	☐	☐
Shooting, stabbing or burning pains, muscle aches	☐	☐	☐
Muscle stiffness or slowness of movement	☐	☐	☐
Headache, neck stiffness or eye pain with eye movements	☐	☐	☐
Loss of the ability to speak or understand what others are saying, slurred speech	☐	☐	☐
Memory problems, searching for words, slowed thinking	☐	☐	☐
Loss of consciousness, reduced awareness, coma	☐	☐	☐
Tremor (shaking)	☐	☐	☐
Muscle twitches or involuntary movements	☐	☐	☐
Seizures	☐	☐	☐
Difficulty with walking or balance	☐	☐	☐
Vision loss in one or both eyes, double vision, blurry vision, trouble focusing	☐	☐	☐
Loss of sense of smell or taste	☐	☐	☐
Tinnitus (ringing in your ears)	☐	☐	☐
Hearing loss	☐	☐	☐
Bowel or bladder problems	☐	☐	☐
Dizziness, spinning, unsteadiness	☐	☐	☐
Trouble with sleep, including falling/staying asleep, poor quality sleep, acting out dreams (talking, kicking, etc.)	☐	☐	☐

POST COVID-19 RECOVERY CLINIC
BASELINE QUESTIONNAIRE

8. PSYCHIATRY SCREEN

Please select an answer for each of the following questions based on what you have been feeling and experiencing during the last 2 weeks.		Clinician Use Only: Item Score	Clinician Use Only: Screen Score
How often have you been feeling nervous, anxious or on edge?	☐ Not at all<br>☐ Several days<br>☐ More than half of the days<br>☐ Nearly every day		☐
How often have you not been able to stop or control your worrying?	☐ Not at all<br>☐ Several days<br>☐ More than half of the days<br>☐ Nearly every day		
How often have you experienced little interest or pleasure in doing things?	☐ Not at all<br>☐ Several days<br>☐ More than half of the days<br>☐ Nearly every day		☐
How often have you been feeling down, depressed or hopeless?	☐ Not at all<br>☐ Several days<br>☐ More than half of the days<br>☐ Nearly every day		
Have you ever felt that you ought to cut down on your drinking or drug use?	☐ Yes<br>☐ No		☐
Have people annoyed you by criticizing your drinking or drug use?	☐ Yes<br>☐ No		
Have you ever felt bad or guilty about your drinking or drug use?	☐ Yes<br>☐ No		
Have you ever had a drink or used drugs first thing in the morning to steady your nerves or to get rid of a hangover?	☐ Yes<br>☐ No		
Have you had frequent unwanted thoughts that seem difficult to control?	☐ Yes ☐ Maybe/Not sure<br>☐ No		☐
Have you felt an urge that was difficult to control to repeat actions (e.g. washing, checking)?	☐ Yes ☐ Maybe/Not sure<br>☐ No		
Have you felt very happy or irritable for time periods lasting at least 2 days?	☐ Yes ☐ Maybe/Not sure<br>☐ No		☐
Have you felt full of energy for time periods lasting at least 2 days?	☐ Yes ☐ Maybe/Not sure<br>☐ No		
Have you thought that other people are plotting against you or are trying to hurt you?	☐ Yes ☐ Maybe/Not sure<br>☐ No		☐
Have you noticed any unusual experiences – like hearing or seeing things other people couldn't or when other people are not present?	☐ Yes ☐ Maybe/Not sure<br>☐ No		

POST-COVID-19 RECOVERY CLINIC
BASELINE QUESTIONNAIRE

9. TRAUMATIC EVENTS

The following questions refer specifically **to ANY TRAUMATIC EXPERIENCES YOU MAY HAVE EVER HAD**, including your hospital stay for COVID-19 disease or any other traumatic event.		Clinician Use only: Item Score	Clinician Use only: Screen Score
**During the last 2 weeks have you:**			
Had nightmares about these events or thought about these events when you did not want to?	☐ Yes ☐ No		☐
Tried hard not to think about these events or went out of your way to avoid situations that reminded you of these events?	☐ Yes ☐ No		
Been constantly on guard, watchful, or easily startled?	☐ Yes ☐ No		
Felt numb or detached from people, activities, or your surroundings?	☐ Yes ☐ No		
Felt guilty or unable to stop blaming yourself or others for these events or any problems the event may have caused?	☐ Yes ☐ No		

10. FATIGUE SEVERITY SCALE

Choose a number from 1 to 7 that indicates how much you agree with the following statements. Please base your answer on how you have been feeling on average over the past week.

1 = strongly disagree 4 = neither agree nor disagree 7 = strongly agree.

**During the past week, I have found that:**	**1**	**2**	**3**	**4**	**5**	**6**	**7**
My motivation is lower when I am fatigued	☐	☐	☐	☐	☐	☐	☐
Exercise brings on my fatigue	☐	☐	☐	☐	☐	☐	☐
I am easily fatigued	☐	☐	☐	☐	☐	☐	☐
Fatigue interferes with my physical functioning	☐	☐	☐	☐	☐	☐	☐
Fatigue causes frequent problems for me	☐	☐	☐	☐	☐	☐	☐
My fatigue prevents sustained physical functioning	☐	☐	☐	☐	☐	☐	☐
Fatigue interferes with carrying out certain duties and responsibilities	☐	☐	☐	☐	☐	☐	☐
Fatigue is among my three most disabling symptoms	☐	☐	☐	☐	☐	☐	☐
Fatigue interferes with my work, family or social life	☐	☐	☐	☐	☐	☐	☐

Thank you for completing this questionnaire!
Please bring it to your upcoming appointment at the Post COVID-19 Recovery Clinic.

Questionnaire reviewed by:

Signature ____________ Printed name ____________ Date ____________
