# Supplementary material for: Associations between demographic, clinical, and socioeconomic factors and mental health in long COVID: A clinic-based cross-sectional study
Source: PLoS One. 2026 Mar 18;21(3):e0342516. doi: 10.1371/journal.pone.0342516 (PMC12998830; doi:10.1371/journal.pone.0342516)
Supplement: S2 File — (DOCX) [file pone.0342516.s002.docx]

Supplementary 2

Table 2. Association between demographics, social determinants of health and clinical history and anxiety among patients with long COVID-19

| **Variable** | **Univariate p-value** | **Multivariable p-value** | **Relative Risk**  **(95% CI)** |
| --- | --- | --- | --- |
| **Age** | 0.001 | 0.005 | 0.902 (0.840-0.969) |
| **Sex** | 0.001 | 0.867 | 1.006 (0.938-1.079) |
| **Live with others vs Live alone** | 0.106 | 0.837 | 0.993 (0.926-1.064) |
| **Private home vs Assisted living** | 0.110 | 0.408 | 1.030 (0.960-1.107) |
| **Employed vs Unemployed** | 0.873 | n/a | n/a |
| **Work full-time vs part-time** | 0.000 | 0.456 | 1.026 (0.960-1.096) |
| **Ethnic** | 0.262 | n/a | n/a |
| **Cognitive Issues** | 0.000 | 0.000 | 1.447 (1.339-1.562) |
| **Sensory Issues** | 0.000 | 0.943 | 0.998 (0.932-1.068) |
| **Physical Issues** | 0.000 | 0.000 | 1.295 (1.141-1.469) |
| **Disabilities** | 0.000 | 0.029 | 1.118 (1.012-1.235) |
| **Chronic conditions** | 0.000 | 0.864 | 1.007 (0.931-1.088) |

Table 3. Association between demographics, social determinants of health and clinical history and depression among patients with long COVID-19

| **Variable** | **Univariate p-value** | **Multivariable p-value** | **Relative Risk**  **(95% CI)** |
| --- | --- | --- | --- |
| **Age** | 0.005 | 0.021 | 0.915 (0.849-0.987) |
| **Sex** | 0.211 | n/a | n/a |
| **Live with others vs Live alone** | 0.287 | n/a | n/a |
| **Private home vs Assisted living** | 0.114 | 0.365 | 1.033 (0.963-1.107) |
| **Employed vs Unemployed** | 0.866 | n/a | n/a |
| **Work full-time vs part-time** | 0.026 | 0.234 | 0.958 (0.892-1.028) |
| **Ethnic** | 0.635 | n/a | n/a |
| **Cognitive Issues** | 0.000 | 0.000 | 1.543 (1.421-1.675) |
| **Sensory Issues** | 0.000 | 0.686 | 1.015 (0.945-1.090) |
| **Physical Issues** | 0.000 | 0.000 | 1.427 (1.236-1.647) |
| **Disabilities** | 0.000 | 0.453 | 1.040 (0.939-1.151) |
| **Chronic conditions** | 0.000 | 0.234 | 1.053 (0.967-1.146) |
